# Insulin-like growth factor 1 receptor (*IGF1R*)-dependent signaling regulates blastocyst formation during early embryonic development

**DOI:** 10.3389/fcell.2026.1783082

**Published:** 2026-02-19

**Authors:** Chi-Hun Park, Young-Hee Jeoung, JiTao Wang, Bhanu P. Telugu

**Affiliations:** Division of Animal Sciences, University of Missouri, Columbia, MO, United States

**Keywords:** base editing, embryotropic factor, IGF1R, lineage specification, pig embryos, preimplantation development

## Abstract

Insulin-like growth factor 1 (IGF1) signaling is a conserved regulator of embryonic growth and survival. However, the specific role of IGF1 signaling mediated by its cognate receptor IGF1R during mammalian preimplantation development remains unclear and unexplored. In this study, we employed both genetic ablation using cytidine deaminase base editors and pharmacological inhibition to assess the role of IGF1R in porcine early embryonic development. Embryos lacking *IGF1R* advanced through early cleavage divisions and progressed to blastocyst formation; however, they displayed delayed blastocyst development and significantly increased apoptosis. Lineage segregation was largely unperturbed. Exogenous IGF-1 supplementation did not ameliorate developmental impairments in *IGF1R*-knockout embryos and instead exacerbated apoptotic responses when receptor signaling was compromised. Collectively, these results establish that IGF1R signaling is dispensable for cell fate specification but is crucial for regulating blastocyst growth dynamics and embryonic viability.

## Highlights

- *IGF1R* is dispensable for early cleavage divisions but is required for timely blastocyst formation.
- *IGF1R* loss delays blastocyst expansion without disrupting lineage segregation.
- Dual IGF1R/INSR inhibition phenocopies *IGF1R* genetic ablation.
- IGF-1 supplementation fails to rescue inhibition and increases apoptotic stress in *IGF1R* knockout embryos.

## Introduction

Early embryonic development in mammals depends on tightly regulated processes, including cleavage divisions, compaction, and cell lineage specification. Regulation of these tightly coordinated events determines the quality of the resulting blastocysts, which is a strong predictor of implantation potential and subsequent fetal development. Therefore, it is imperative to elucidate the endogenous pathways that support growth and survival at this stage. The insulin-like growth factor (IGF) system is among the most evolutionarily conserved regulators of early embryo development. The insulin-like growth factor (IGF) system consists of two ligands (IGF-I and IGF-II), multiple receptors including the IGF1R, the insulin receptor fetal isoform (INSR-A), and the IGF2R, which functions primarily as a scavenger receptor for IGF-II, as well as IGF-binding proteins that modulate ligand availability and signaling dynamics (references; LeRoith; Scalia) (Toropainen et al., 1995; LeRoith et al., 2021; Scalia et al., 2023). IGF-I is synthesized by reproductive tissues such as the ovary, oviduct, follicular fluid, and uterine secretions, resulting in a physiologically IGF-rich environment for the preimplantation embryo (Oosterhuis et al., 1998; Oliver et al., 1989). Multiple mammalian species, including mice, cats, cattle, pigs, and humans, express IGF ligands and their cognate receptors, IGFIR and IGF2R, during the preimplantation period, potentially supporting conserved paracrine and autocrine functions (Lighten et al., 1997; Smotrich et al., 1996).

The IGF-I and IGF1R signaling primarily regulate proliferation, metabolism, and anti-apoptotic pathways through IRS1/2-dependent activation of PI3K/AKT signaling, as well as MAPK/ERK and PLC pathways (Ipsa et al., 2019; Harvey and Kaye, 1992). These signaling cascades converge on downstream mediators such as S6K1, GSK3, and GLUT4/8, thereby influencing cell growth, glucose homeostasis, glycogen synthesis, and resistance to apoptosis (Shiota et al., 2003; Becker et al., 2011). For example, supplementation of human embryo culture medium with IGF-1 at 13 ng/mL after *in vitro* fertilization markedly increased blastocyst formation and reduced apoptosis (Lighten et al., 1998). Similarly, IGF-I supplementation has been shown to consistently improve developmental competence across species by enhancing blastocyst formation, increasing total cell numbers, reducing apoptosis, and improving metabolic fitness (Spanos et al., 2000; Sirisathien et al., 2003; Fernandez-Gonzalez et al., 2021; Green and Day, 2013). A recent mechanistic study demonstrated that IGF-1 activates Wnt/β-catenin signaling to drive TE proliferation in pig embryos (Kim et al., 2025). Collectively, these findings highlight the critical role of IGF1 signaling in supporting blastocyst growth.

Although IGF supplementation has been extensively studied, the effects of IGF1R in mediating canonical IGF1 signaling remain poorly understood, particularly in large animals. In pigs, IGF1R is expressed throughout preimplantation development, a pattern that more closely resembles that of bovine and human embryos than of mouse embryos, where *Igf1r* expression appears later at the 8-cell stage (Riley et al., 2005). In mice, *Igf1r* is essential for preimplantation development, as *Igf1r*-deficient embryos fail to progress beyond the cavitation stage (Bedzhov et al., 2012). *IGF1R*-null pigs generated using CRISPR/Cas9 exhibit severe growth restriction and neonatal lethality, underscoring the essential role of *IGF1R* in somatic growth. However, its potential role in early embryogenesis remains unexplored (Nagaya et al., 2024). The main goal of this study was to investigate the role of IGF1R-mediated signaling in early embryonic development using pig embryos that closely mimic human, bovine, and other livestock species in receptor expression and developmental dynamics.

## Materials and methods

All experiments were performed as per the approved guidelines of the University of Missouri, Institutional Biosafety Committee, and Institutional Animal Care and Use Committee protocol # 45081. All chemicals were obtained from Sigma-Aldrich (St. Louis, MO) unless otherwise specified.

### Generation of *IGF1R* KO somatic cells

**FIGURE 1:**
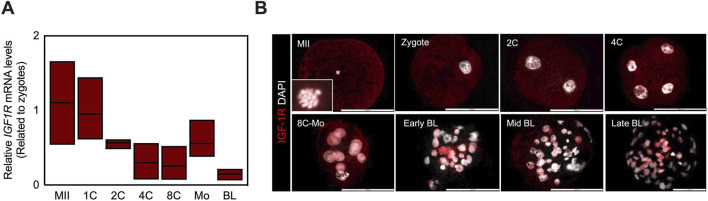
IGF1R expression in preimplantation pig embryos. **(A)** IGF1R expression in pig oocytes and embryos during preimplantation development. Embryos generated by IVF were cultured for 7 days. YWHAZ served as the internal control. n = 5 embryos in every sample. The experiment was repeated three times. Data are presented as mean values. The relative mRNA level of IGF1R in oocytes is defined as 1.0. **(B)** Representative fluorescence images of localization of IGF1R (red) in pig oocyte and embryos from zygote to the blastocyst stage. Images are representative of at least 7-10 embryos per stage. Nuclei were counterstained with 4,6-diamidino-2-phenylindole (DAPI, grey). Scale bar represents 100 μm. MII; matured oocyte at metaplate II, Zy; zygote, Mo; morula, BL; blastocyst.

Two candidate single guide (sg) RNAs targeting exon 2 (E2-1 and E2-2) and one targeting exon 5 (E5) of the porcine *IGF1R* gene were designed using BE-Designer online software (http://www.rgenome.net) and commercially synthesized by Synthego (Figure 1A; Supplementary Table S1). The BE4 mRNA was generated by *in vitro* transcription of pCMV-BE4-RrA3F plasmid (a gift from Nicole Gaudelli (Addgene plasmid #138340; http://n2t.net/addgene:138340; RRID:Addgene_138340)) using the mMESSAGE mMACHINE T7 kit (Invitrogen) according to the manufacturer’s instructions. Following transcription, the mRNA was purified by ethanol precipitation. Candidate sgRNAs were co-transfected with BE4 mRNAs into porcine fetal fibroblasts (PFF) by nucleofection (Lonza), followed by culture in 10-cm dishes at a seeding density of 5 cells/cm^2^ to generate single-cell colonies as per established and published protocols (Simpson et al., 2024; Park et al., 2022; Park et al., 2017). After 10–14 days, single-cell colonies were isolated and genotyped by Sanger sequencing (Supplementary Table S2).

### Screening of editing outcomes

Genomic DNA from candidate PFF clones was isolated by lysing cells using lysis buffer (40 nM Tris-HCl, 1% Triton X-100, 1% NP-40, and 0.4 ng/mL proteinase K) at 55 °C for 1 h and 95 °C for 10 min. PCR amplification was performed using either KOD Hot Start master mix (Novagen) or Bioline Taq with the following cycling conditions: initial denaturation and polymerase activation at 95 °C for 3 min, followed by 35 cycles of 95 °C for 20 s, 60 °C for 10 s, and 70 °C for 10 s, with a final extension at 70 °C for 5 min. PCR products were purified using the NucleoSpin gel and PCR clean-up kit (Machery Nagel) and subjected to Sanger sequencing. The primers used were provided in Supplementary Table S2. Targeted genomic sites were amplified using Phusion polymerase (Thermo Fisher Scientific). Paired-end sequencing of PCR amplicons was conducted on an Illumina MiSeq platform by Novogene Bioinformatics Technology Co. Ltd. Potential off-target sites for each sgRNA were predicted using Cas-OFFinder (http://www.rgenome.net/cas-offinder/) to analyze site-specific edits. For the off-target assay, up to three base mismatches and no DNA or RNA bulges were permitted.

### Generation of *IGF1R* wild-type and knockout porcine embryos

Cumulus-oocyte complexes (COCs) were obtained from De Soto Biosciences (Seymour, TN, United States). We generated *IGF1R* wild-type (WT) and knockout (KO) embryos by somatic cell nuclear transfer (SCNT) using WT and KO PFFs. The WT and KO PFFs were synchronized to the G1/G0 phase by serum deprivation (DMEM with 0.1% FBS) for 96 h. Oocytes were enucleated by aspirating the polar body and MII metaphase plates with a micropipette (Humagen, Charlottesville, VA, United States) in 0.1% DPBS supplemented with 5 μg/mL cytochalasin B. Following enucleation, a single donor cell was placed into the perivitelline space of each enucleated oocyte. Cell-oocyte couplets were fused by applying two direct current pulses (1-s interval) of 2.0 kV/cm for 30 microseconds using an ECM 2001 Electroporation System (BTX, Holliston, MA). After fusion, reconstructed zygotes were activated by a direct current pulse of 1.0 kV/cm for 60 microseconds, followed by post-activation in 2 mM 6-dimethylaminopurine for 3 h (Park et al., 2021). After overnight culture in PZM3 medium containing the histone deacetylase inhibitor Scriptaid (0.5 μM), reconstructed zygotes were cultured in PZM3 under low oxygen conditions (5% O_2_ and 5% CO2 in 90% N2) (Park et al., 2021).

### Live caspase-3/7 staining

Apoptotic activity in embryos was assessed using a live caspase-3/7 detection assay (Invitrogen). Embryos were incubated in culture medium supplemented with the caspase-3/7 reagent at a final dilution of 1:400 for 30 min at 38.5 °C under standard culture conditions. Following incubation, embryos were washed briefly in fresh medium and immediately imaged using fluorescence microscopy.

### OSI-906 dose titration and stage-specific treatment

To determine dynamic range and a non-toxic working concentration of OSI-906 (OSI), a dual IGF1R/INSR tyrosine kinase inhibitor (Mulvihill et al., 2009), presumptive zygotes were cultured continuously from the one-cell stage to the blastocyst stage in PZM-3 medium supplemented with OSI-906 at final concentrations of 0, 0.001, 0.01, 0.1, 1, or 10 µM. In the second experiment, embryos that reached the morula stage were randomly assigned to treatment groups and cultured in PZM-3 medium supplemented with OSI-906 at concentrations of 0, 0.1, 1, 2, 5, or 10 µM. Media were refreshed daily with freshly prepared inhibitor. Embryos were maintained under standard culture conditions, and media containing freshly prepared OSI-906 were replaced every 24 h. Cleavage rates were assessed at the 2-4 cell stage (48 h post-activation), and blastocyst formation was evaluated on day 7 (168 h).

### Expression analysis

RNA isolation and cDNA synthesis were performed as previously described (Sheets et al., 2018). Primers listed in Supplementary Table S3 were used for qRT-PCR. Early embryos were rinsed three times with 0.1% PBS/polyvinylpyrrolidone and fixed with 4% paraformaldehyde in PBS for 5 min. Samples were permeabilized with 0.5% Triton X-100/PBS for 30 min, then blocked for 2 h with a buffer containing 5% bovine serum albumin and 0.1% Triton X-100 in PBS. Samples were incubated with primary antibodies overnight at 4 °C, followed by treatment with secondary antibodies for 1 h. Nuclear DNA was counterstained with DAPI (Thermo Fisher Scientific) for 5 min. Samples were mounted and imaged using a Leica DM 4000B microscope and the Leica Application Suite Advanced Fluorescence software (Leica Microsystems, Wetzlar, Germany). All antibody information is provided in Supplementary Table S4.

### Statistical analysis

All quantitative data are presented as mean ± SEM unless otherwise indicated. Statistical analyses were performed using GraphPad Prism 9 (GraphPad Software, San Diego, CA, United States). For comparisons between two groups, unpaired two-tailed Student’s t-tests were applied when data satisfied assumptions of normality and equal variance. For comparisons among multiple groups, one-way ANOVA followed by Dunnett’s multiple comparisons test was used to assess differences relative to the control group, as indicated in the figure legends. Developmental rates and proportions (e.g., blastocyst formation rate and percentage of caspase-3 positive embryos) were analyzed using the same statistical framework. Each experiment was performed using at least three independent biological replicates unless otherwise stated, and the number of embryos analyzed per group is provided in the corresponding figure legends. Differences were considered statistically significant when *P* < 0.05.

## Results

### Dynamic expression and localization of IGF1R during preimplantation development

To elucidate the developmental context of IGF1R function, the expression pattern was examined during porcine preimplantation development. Quantitative RT-PCR analysis revealed a canonical maternal-to-zygotic expression pattern: *IGF1R* transcripts were abundant in oocytes, decreased progressively through the 4- to 8-cell stages, increased again at the morula stage, and declined again in blastocysts. This temporal pattern coincided with compaction and blastocoel formation, suggesting stage-specific regulation of *IGF1R* expression (Figure 1A). Immuno-fluorescence analysis demonstrated dynamic changes in IGF1R localization (Figure 1B). During early cleavage stages, IGF1R protein was predominantly cytoplasmic. From the late 8-cell stage onward, the IGF1R signal became detectable within the nucleus, and by the blastocyst stage, a subset of embryos exhibited punctate intranuclear enrichment consistent with nucleolar localization. In mid-to late-stage blastocysts, IGF1R was further concentrated within a subset of nuclei as distinct punctate foci. These observations indicate that IGF1R undergoes regulated spatial redistribution during preimplantation development, although the functional significance of this redistribution at this stage remains unresolved.

### Efficient generation of *IGF1R* knockout fibroblasts and SCNT-derived embryos

**FIGURE 2:**
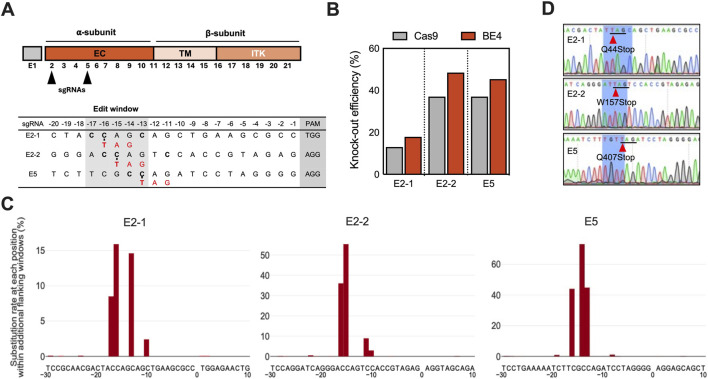
Generating *IGF1R* Knockout fibroblast lines using Cas9 or cytidine base editor (BE4). **(A)** Schematic of the structure of the porcine *IGF1R* encoded by exons 1-21 (E1-21), which consists of an extracellular (EC) α subunit and a β subunit composed of a transmembrane (TM), an intracellular tyrosine kinase (ITK) domain, and a C-terminal tail. Candidate sgRNAs were marked by the black arrowhead. Target-site sequences within the *IGF1R* loci. Cytosines (C) in the editing window relative to the PAM region were highlighted in blue and grey boxes, respectively. **(B)** The knockout efficiency of Cas9 and BE4 at the target sites in fibroblasts. Targeted conversion of C-to-T within the editing window results in translational stop codon and an inactive gene product. Amp-seq assessed the cells for BE4 and by Inference of CRISPR Edits (ICE) analysis using Sanger data for Cas9. **(C)** Mutation spectra of the three *IGF1R* target sites analyzed by deep sequencing. Frequencies of nucleotide substitutions at each position from–17 to −9 bp relative to the target PAM sequence were plotted. Negative area of the y-axis control data. **(D)** Chromatograms of PCR amplicons spanning the *IGF1R* gRNA target sites are shown for single-cell clones. Red arrowheads mark the targeted cytosine that was converted to thymidine to generate stop codons (underlined in black).

To investigate the potential role of IGF1R, a functional KO of the porcine *IGF1R* gene was generated using the fourth-generation cytidine base editor (BE4). Candidate sgRNAs were designed to target the extracellular domain of *IGF1R* within exon 2 (E2-1, E2-2) and exon 5 (E5-1), positioning the catalytic cytidines within the BE4 editing window (N13-N17 upstream of the PAM). BE4-mediated conversion of C-to-T at these positions was expected to introduce premature termination codons (PTCs) and functionally inactivate the gene (Figure 2A) (Yu et al., 2020). Transfection of porcine fetal fibroblasts (PFFs) with BE4 mRNA and sgRNAs resulted in efficient cytidine deamination, as confirmed by targeted amplicon sequencing (Amp-seq). Editing efficiencies varied by sgRNA, yielding 17% (E2-1), 56% (E2-2), and 73% (E5-1) conversion, with E5-1 showing the highest efficiency (Figure 2B; Supplementary Table S5). Other editing outcomes, including imperfect and minimal indel formation (0.69%–1.03%), were also detected (Figure 2C; Supplementary Figure S1). For comparison, SpCas9 editing with the same sgRNAs yielded lower overall efficiencies (13%, 37%, and 37%, respectively) (Figure 2B) and resulted in a heterogeneous outcome, including both in-frame and frameshift mutations (Supplementary Table S1; Supplementary Figure S5). In contrast, BE4 editing predominantly yielded precise PTC-inducing substitutions, demonstrating higher accuracy, albeit guide-dependent (Figure 2C). Single-cell colonies derived from the edited PFFs were expanded and sequenced, revealing that 9 of 24 clones carried the intended point mutations introducing the PTC. Specifically, 2 of 12 (16.7%) E2-1 colonies harbored the Q44* mutation, 2 of 5 (40%) E2-2 targeted colonies carried the W157* mutation, and 5 of 7(85.7%) E5-1 targeted colonies contained the Q407* mutation (Figure 2D). All edited colonies were homozygous for the introduced PTCs, except for a single heterozygous E2-1 clone (Supplementary Figure S1B). Sanger sequencing confirmed precise C-to-T substitutions generating the corresponding stop codons, with no off-targeting events detected. A homozygous *IGF1R* KO clone (E2-4) and the precursor PFF were selected as nuclear donors for SCNT to generate KO and WT control embryos, respectively.

### Loss of *IGF1R* delays blastocyst development but not early cleavage events

**FIGURE 3:**
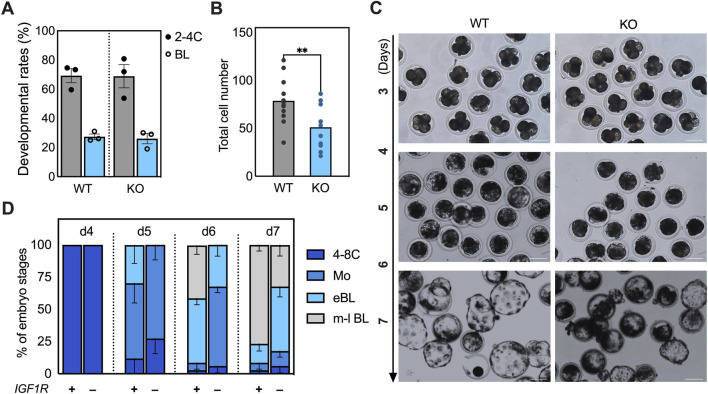
Functional inactivation of *IGF1R* leads to a delay in blastocyst development. **(A)** Developmental rate of WT and KO embryos. On day (D) 3 and 7 post-activation, the percentage of embryos that developed to the 2-4 cell and blastocyst stage was recorded. **(B)** The dot plot shows the number of cells in individual blastocysts. Blastocysts at D7 were fixed and stained with DAPI to count the number of cells within each blastocyst. Average cell numbers between WT (n = 12) and KO (n = 11) blastocysts were compared using an unpaired t-test. ** indicate statistical differences (p < 0.01) **(C)** Images of WT and KO embryos after selection that reached the morula-, and early to expanded blastocyst stages from D3 to D7 culture *in vitro*. Four-cell-stage embryos generated from WT or KO cells were selected, and their kinetics were tracked at different time points. Scale bar, 100 μm. **(D)** The percentage of 4 to 8-cell embryos (c) that reached the morula and early-to late blastocyst stages at D4, 5, 6, and 7 was calculated and presented as the developmental rate. The experiment was repeated at least three times.

The functional requirement for IGF1R during early development was assessed by comparing developmental kinetics between *IGF1R* WT and KO SCNT embryos. Cleavage rates and overall blastocyst formation rates were indistinguishable between the two groups, indicating that *IGF1R* is dispensable for early mitotic divisions and blastocyst formation (Figure 3A). Despite similar blastocyst rates, KO blastocysts contained significantly fewer total cells than WT controls (p < 0.01; Figure 3B). Time-course analysis showed that while 85.4% of WT morulae had progressed to early blastocysts by day 5 (Figure 3C), the majority (73.4%) of KO embryos remained at the morula stage at this time point. By day 6, 91% of WT embryos predominantly reached early to mid-blastocyst stages, whereas only a minority (32.2%) of KO embryos did. Although most KO embryos (82.2%) eventually formed blastocysts by day 7, fewer (32.2%) progressed to expanded or hatching stages (Figures 3C,D), indicating that KO embryos displayed a pronounced delay in developmental kinetics.

### *IGF1R* deficiency preserves lineage allocation despite altered developmental kinetics

**FIGURE 4:**
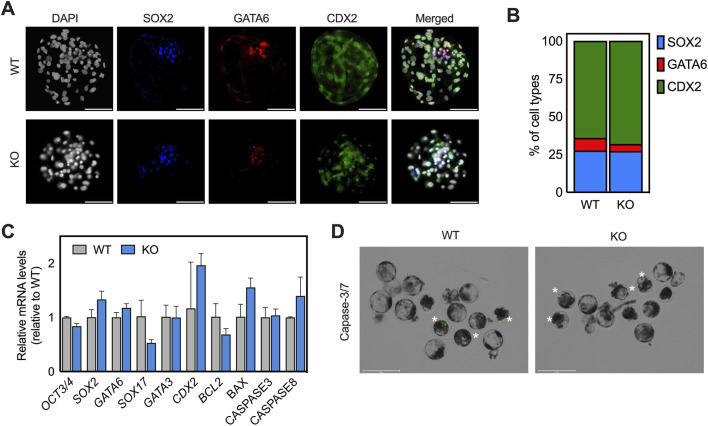
*IGF1R* deficiency delays blastocyst growth and increases apoptotic susceptibility without disrupting lineage segregation. **(A)** Representative immunofluorescence images of key lineage markers in day 7 (D7) blastocysts. CDX2 marks trophectoderm (green), SOX2 marks epiblast (cyan), and GATA6 marks primitive endoderm (PrE; red). Nuclei were counterstained with DAPI (blue). Images are representative of at least five embryos per group. Scale bar, 100 μm. **(B)** Quantification of lineage composition in WT and KO blastocysts based on immunostaining in (A). The proportion of CDX2+, SOX2+, and GATA6+ cells was calculated relative to total cell number per blastocyst. **(C)** Relative transcript abundance of genes associated with lineage specification and developmental progression in WT and KO blastocysts. Expression levels were normalized to internal controls, and WT values were set to 1. Error bars indicate SEM. **(D)** Representative images of live caspase-3/7 staining (green) in WT and KO blastocysts at D7. Asterisks indicate caspase-3 positive (CS3+) cells. Scale bar: 275 μm.

The impact of growth delay on early lineage specification was assessed by examining the expression of key transcription factors associated with TE, epiblast (EPI), and primitive endoderm (PrE) identities. Immunofluorescence confirmed the presence of CDX2-positive TE, SOX2-positive EPI, and SOX17-positive PE in both groups, with no detectable differences in lineage proportions (Figure 4A). Despite the defects in blastocyst development, lineage allocation remained largely intact. Quantitative analysis revealed more subtle shifts consistent with developmental delay rather than fate disruption (Figure 4B). *SOX2* transcripts were reduced in KO blastocysts relative to WT embryos, whereas *CDX2* expression was modestly elevated. *GATA6* expression was largely preserved (Figure 4C). Modest reductions in hypoblast-associated transcripts (*SOX17*) were observed in some KO samples (Figure 4C). Collectively, these data indicate that IGF1R primarily regulates blastocyst growth kinetics and expansion, while lineage specification and spatial segregation remain intact in its absence.

### *IGF1R* loss increases apoptotic susceptibility in delayed blastocysts

The association between impaired blastocyst development in KO embryos and increased cell death was evaluated using live caspase-3/7 staining and transcript analysis. During early cleavage stages, Occasional caspase-3/7-positive (CS+) signals were detected; however, these were spatially restricted to small punctate structures consistent with the extruded polar bodies and were not associated with delayed or degenerated embryos. Importantly, caspase-positive blastomeres were not detected during cleavage stages, indicating that *IGF1R* loss does not induce apoptosis during early embryonic divisions (Supplementary Figure S2A). However, CS + cells were detected more frequently in KO blastocysts compared with WT controls, particularly in embryos exhibiting delayed development or abnormal morphology (Figure 4D). Quantitative analysis showed a higher percentage of CS + blastocysts in the KO group, although this increase did not reach statistical significance (p = 0.1397; Supplementary Figure S2B). At the transcriptional level, KO blastocysts exhibited reduced expression of the anti-apoptotic gene *BCL2* and increased expression of pro-apoptotic genes *BAX* and *CASPASE8*, consistent with heightened apoptotic susceptibility (Figure 4C). These findings indicate that loss of *IGF1R* compromises blastocyst survival capacity, particularly under conditions of delayed developmental progression.

### Dual IGF1R/INSR inhibition by OSI-906 phenocopies *IGF1R*-null developmental defects

**FIGURE 5:**
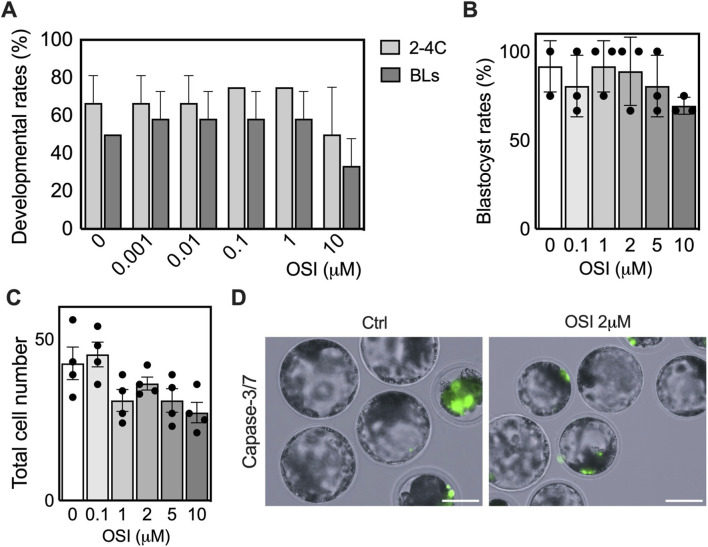
Dual IGF1R/INSR inhibition by OSI-906 recapitulates the *IGF1R*-null phenotype. **(A)** Developmental competence of embryos treated with increasing concentrations of OSI-906 from the one-cell stage. Bars indicate cleavage rates assessed at the 2-4 cell stage (48 h post-activation), and blastocyst formation rates estimated at 168 h. **(B)** Blastocyst formation rates following OSI-906 treatment initiated at the morula stage. Data are presented as the percentage of embryos reaching the blastocyst stage relative to the number of morulae at treatment onset. Scale bar: 150 μm. **(C)** Total cell number of blastocysts at D7 following OSI-906 treatment initiated at the morula stage. **(D)** Representative images of live caspase-3/7 staining (green) in control (ctrl) and OSI-906 (2 μM) blastocysts at D6. Asterisks indicate CS3+ cells. Increased caspase-3 activity was primarily observed in developmentally delayed or morphologically abnormal embryos.

To account for potential compensatory signaling through INSR, OSI-906 (OSI), a dual IGF1R/INSR tyrosine kinase inhibitor, was used to inhibit IGF1R and INSR simultaneously across distinct developmental windows and dose ranges. Zygotes were treated continuously with OSI from the one-cell stage through blastocyst formation using concentrations ranging from 0.001 to 10 μM (Figure 5A; Supplementary Figure S3). Across this range, early cleavage to the 2-4 cell stage was largely unaffected at doses up to 1 μM, whereas blastocyst formation was modestly reduced only at the highest concentration tested (10 μM), indicating a broad tolerance to dual receptor inhibition during early cleavage stages. To assess the requirement for IGF1R/INSR signaling during blastocyst maturation more precisely, OSI was next applied specifically during the morula-to-blastocyst transition using a refined dose range (0.1, 1, 2, 5, and 10 μM; Figure 5B). Under these conditions, OSI treatment resulted in delayed blastocyst formation, reduced progression to expanded stages (Figure 5C), and increased apoptosis (Figure 5D), closely resembling the phenotype observed in *IGF1R* KO embryos. Increasing OSI concentrations did not induce more severe developmental impairment beyond this delay, nor did they cause widespread developmental arrest at doses ≤2 μM. Dual IGF1R/INSR inhibition did not worsen developmental outcomes compared with *IGF1R* genetic ablation alone, suggesting that INSR signaling does not play a dominant role during porcine preimplantation development under these conditions.

### IGF-1 supplementation fails to rescue OSI-induced defects and exacerbates apoptotic responses

**FIGURE 6:**
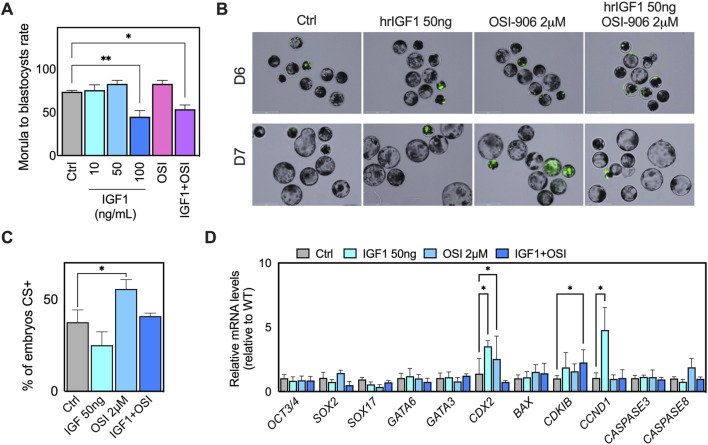
IGF-1 supplementation does not rescue OSI-induced defects during morula-to-blastocyst transition. **(A)** Representative brightfield images of embryos treated at the morula stage with IGF-1 (10, 50, and 100 ng/mL), OSI-906 (2 μM), or the combination of IGF-1 (50 ng/mL) and OSI-906 (2 μM), compared with untreated controls. **(B)** Representative images of live caspase-3 staining (green) in control (ctrl) and treated blastocysts at D6 and D7. CS3+ cells were predominantly observed in embryos exhibiting delayed development or abnormal morphology. Scale bar: 275 μm. **(C)** Quantification of CS3+ blastocysts across treatment groups. Statistical significance was assessed using Dunnett’s multiple comparisons test relative to WT controls. **(D)** Relative transcript abundance of genes associated with lineage specification, cell-cycle regulation, and apoptosis in blastocysts from each treatment group. Expression levels were normalized to internal controls, and WT values were set to 1. Error bars indicate SEM.

IGF-1 supplementation was used as a functional probe to test whether increased ligand input could compensate for impaired IGF1R/INSR-PI3K pathway capacity during the morula-to-blastocyst transition by treating morula-stage embryos with increasing concentrations of IGF-1 alone or in combination with OSI. IGF-1 supplementation alone exerted a dose-dependent effect on blastocyst formation (Supplementary Figure S4). Low to moderate IGF1 concentrations (10–50 ng/mL) modestly increased developmental efficiency, whereas high-dose IGF-1 (100 ng/mL) significantly reduced blastocyst formation compared with control embryos (Figure 6A). Combined treatment with IGF-1 (50 ng/mL) and OSI (2 µM) failed to restore normal blastocyst development. Morula-to-blastocyst rates in the combined treatment group remained significantly lower than those of controls. They were not improved relative to OSI alone (Figure 6A), indicating that ligand supplementation cannot bypass receptor-level inhibition. Consistent with prior work showing that IGF1-driven blastocyst responses are PI3K-dependent and blocked by IGF1R neutralization (Green et al., 2015), ligand supplementation did not overcome receptor-level inhibition in our system.

Apoptosis was assessed by caspase-3/-7 staining, which revealed a significant increase in apoptotic embryos following OSI treatment (p = 0.0113), whereas IGF-1 treatment alone did not differ significantly from controls (Figures 6B,C). The combined IGF-1 + OSI group exhibited intermediate levels of CS + embryos, not statistically different from the control, indicating partial attenuation rather than rescue of OSI-induced apoptotic stress. Apoptotic CS + cells were predominantly confined to small, poorly expanded, or developmentally delayed embryos. At the same time, well-expanded blastocysts at D7 across all groups were largely CS negative (Figure 6B) (Green et al., 2015). Gene expression analysis further supported these phenotypic findings. OSI-906 treatment was associated with marked suppression of *SOX17* and dysregulation of cell-cycle regulators, including increased *CDKN1B* and altered *CCND1* expression, whereas IGF-1 supplementation alone did not restore normal expression profiles (Figure 6D). IGF-1 exposure in the context of receptor inhibition does not compensate for impaired signaling and may exacerbate pathway imbalance. Collectively, these findings indicate that IGF-1 supplementation at the morula stage does not confer developmental benefit and may exacerbate stress responses when receptor signaling is compromised.

## Discussion

The results indicate that IGF1R is not necessary for early cleavage or lineage allocation but is essential for robust proliferation and blastocyst expansion. *IGF1R*-deficient embryos reached the blastocyst stage with significant delays and reduced total cell numbers, demonstrating a quantitative rather than an absolute requirement for IGF1R in growth. The defects observed in KO embryos are consistent with the established roles of IGF1R in activating IRS1/2-dependent PI3K/AKT and MAPK/ERK pathways, which regulate cell cycle progression, glucose uptake, protein synthesis, and resistance to apoptosis (Ipsa et al., 2019). The reduced blastocyst size and total cell number in *IGF1R*-deficient embryos closely resemble the growth-restricted phenotype of IGF1R-KO pigs *in vivo* (Nagaya et al., 2024), indicating that IGF1R-dependent signaling is required continuously from preimplantation development through postnatal growth.

The levels of *IGF1R* transcript decline during early cleavage and increase at the morula stage, coincidental with compaction and blastocoel formation. These developmental stages involve increased metabolic demand, fluid transport, and coordinated cell proliferation, processes previously shown to be dependent on IGF-PI3K-AKT signaling (Riley et al., 2006). These findings support a model in which IGF1R primarily regulates developmental tempo rather than early embryonic viability. This separation of cleavage competence from blastocyst formation aligns with previous reports in mouse and bovine embryos, where IGF signaling predominantly influences post-compaction development rather than early mitotic divisions (Lighten et al., 1997; Bedzhov et al., 2012). The absence of increased apoptosis during cleavage stages further indicates that IGF1R signaling is dispensable for early embryonic survival. Consistent with this, apoptotic signals in IGF1R-deficient embryos emerged predominantly at the blastocyst stage.

Unlike in mice, where *Igf1r* KO results in preimplantation arrest, porcine *IGF1R* KO embryos were able to cavitate and typically specify TE, EPI, and PrE. This divergence likely reflects species-specific differences in *IGF1R* expression timing and metabolic dependence. Porcine embryos express *IGF1R* continuously from the zygote stage onward, whereas mouse embryos initiate *Igf1r* expression later, making them potentially more sensitive to loss of function (Bedzhov et al., 2012). The potential for compensatory INSR signaling to mitigate *IGF1R* loss was also examined. Dual inhibition of IGF1R and INSR with OSI closely replicated the effects of *IGF1R* genetic ablation across developmental kinetics, blastocyst expansion, and apoptotic susceptibility. Notably, dual inhibition did not exacerbate defects beyond those observed in KO embryos, suggesting that INSR signaling is largely dispensable during porcine preimplantation development.

Despite delayed blastocyst development, *IGF1R*-null embryos maintained proper lineage segregation. This observation is consistent with genetic and pharmacological studies in mouse embryos, which demonstrate that lineage allocation is immune to growth perturbations and signaling delays, provided embryos reach the appropriate developmental stage (Bedzhov et al., 2012; Kang et al., 2013). We explicitly acknowledge that IGF-II–mediated autocrine signaling, potentially acting through IGF1R and/or INSR-A, may modulate the phenotypic penetrance of IGF1R loss during preimplantation development; however, dissecting ligand-specific competition and receptor usage lies beyond the scope of the present study. At the transcriptional level, reduced *SOX17* expression and modestly elevated *CDX2* expression were observed in KO blastocysts, while *GATA6* expression remained largely intact. Given the developmental delay in KO embryos, these transcriptional changes likely reflect stage heterogeneity rather than direct impairment of lineage specification. Reduced *SOX17* expression has been repeatedly associated with delayed or poor-quality blastocysts across mammalian species, including mouse, bovine, and human embryos (Artus et al., 2011; Kuijk et al., 2012). Thus, IGF1R and IGF signaling appear to contribute to reaching the developmental window during which hypoblast segregation and blastocyst development occur, rather than being strictly required for lineage allocation.

IGF signaling suppresses apoptosis through AKT-mediated inhibition of pro-apoptotic pathways, and loss of IGF1R likely lowers the apoptotic threshold during blastocyst development. In our study, apoptotic signals were primarily observed in small or poorly expanded blastocysts, whereas well-expanded blastocysts were predominantly caspase-3-negative. Similar associations between delayed development and increased apoptosis have been reported in bovine and mouse embryos exposed to suboptimal culture conditions or growth factor imbalance (Green and Day, 2013; Velazquez et al., 2011). Notably, recent studies in human embryos have shown that receptor-specific perturbation of IGF1R signaling selectively impairs proliferation and survival programs while largely preserving cell identity (Wamaitha et al., 2020), supporting the interpretation that *IGF1R* loss compromises survival capacity primarily under conditions of delayed developmental progression.

Exogenous IGF-I supplementation enhanced blastocyst development, consistent with embryotropic effects reported across multiple species (Makarevich and Markkula, 2002; Spanos et al., 2000; Sirisathien et al., 2003; Lighten et al., 1998; Choi et al., 2024). Recent evidence that IGF-1 activates Wnt/β-catenin signaling to promote TE proliferation in pigs provides a mechanistic basis for these observations (Kim et al., 2025). However, IGF1 supplementation can either stabilize or destabilize IGF1R signaling depending on the developmental context (Green and Day, 2013).

In our study, exogenous IGF-1 supplementation did not improve blastocyst development in *IGF1R*-deficient embryos and instead increased apoptotic signals, indicating that enhanced ligand availability cannot compensate for receptor-level signaling constraints in this context. This outcome is consistent with observations in human preimplantation embryos, where manipulation of IGF-dependent survival pathways, including IGF-1 supplementation and PI3K inhibition, does not uniformly enhance blastocyst development and is frequently associated with increased apoptosis in developmentally delayed or compromised embryos (Green et al., 2015). These findings support the interpretation that IGF-1 treatment functions as a probe of signaling competence rather than a physiological rescue strategy, and that elevated apoptosis reflects developmental stress associated with delayed blastocyst development.

## Conclusion

Collectively, these findings establish IGF1R as a key regulator of blastocyst growth kinetics, survival capacity, and developmental tempo in the porcine embryo. Although IGF1R is not required for lineage specification, its absence delays progression through critical developmental transitions, increases apoptotic susceptibility, and limits blastocyst expansion.

## Data Availability

The original contributions presented in the study are included in the article/Supplementary Material, further inquiries can be directed to the corresponding author.
