# Supplementary material for: Insulin-like growth factor 1 receptor (IGF1R)-dependent signaling regulates blastocyst formation during early embryonic development
Source: Front Cell Dev Biol. 2026 Feb 19;14:1783082. doi: 10.3389/fcell.2026.1783082 (PMC12960595; doi:10.3389/fcell.2026.1783082)
Supplement: Supplementary file 1 [file DataSheet1.pdf]

Supporting Information

Insulin-like Growth Factor 1 Receptor (*IGF1R*)-Dependent Signaling Regulates Blastocyst Formation During Early Embryonic Development

Chi-Hun Park[1†], Young-Hee Jeoung[1†], JiTao Wang[1], and Bhanu P. Telugu[1*]

[1] Division of Animal Sciences, University of Missouri, Columbia, MO 65211.

*Correspondence: telugub@umsystem.edu

This PDF file includes:

Supplementary Figures (1 to 4)
- sFigure 1. Base-editing efficiency and mutation profiles at the IGF1R locus.
- sFigure 2. Increased apoptotic activity in IGF1R-deficient blastocysts.
- sFigure 3. OSI-906 dose titration in parthenogenetic embryos.
- sFigure 4. Quantification of blastocyst formation kinetics following pharmacologic perturbation.

Supplementary Tables (1-5)
- sTable 1. The genotyping primer sequence of IGF1R
- sTable 2. The sgRNA sequence of *IGF1R*
- sTable 3. RT-qPCR primers
- sTable 4. Antibody information
- sTable 5. Summary of the CRISPR-induced mutations

Supplementary Figures

a

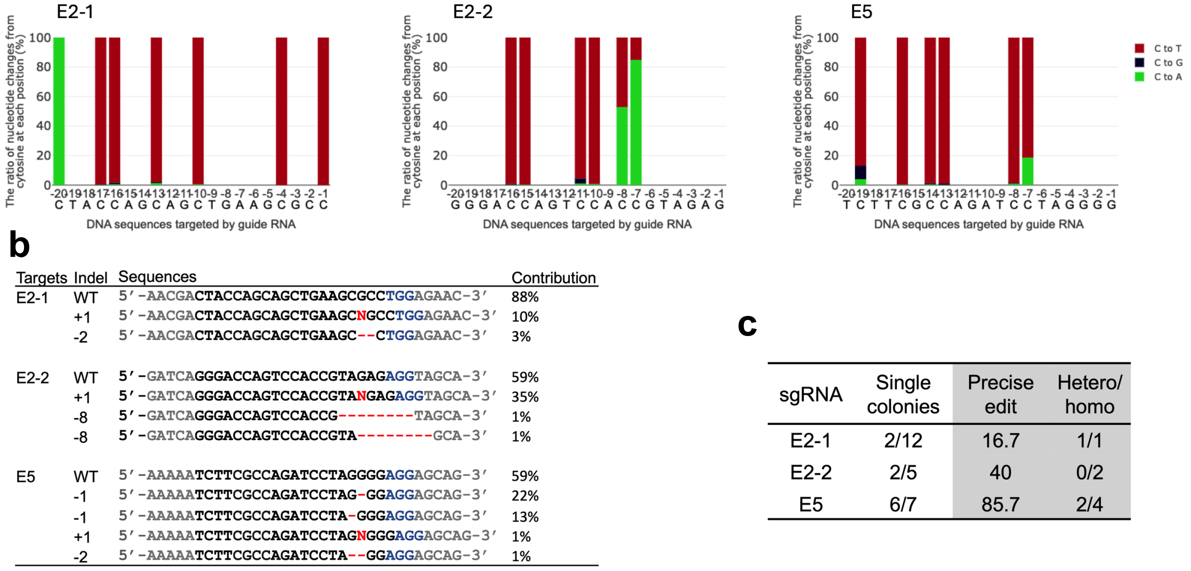

b

Targets	Indel	Sequences	Contribution
E2-1	WT	5′-AACGACTACCAGCAGCTGAAGCGCCTGGAGAAC-3′	88%
	+1	5′-AACGACTACCAGCAGCTGAAGCNGCCTGGAGAAC-3′	10%
	-2	5′-AACGACTACCAGCAGCTGAAGC--CTGGAGAAC-3′	3%
E2-2	WT	5′-GATCAGGGACCAGTCCACCGTAGAGAGGTAGCA-3′	59%
	+1	5′-GATCAGGGACCAGTCCACCGTANGAGAGGTAGCA-3′	35%
	-8	5′-GATCAGGGACCAGTCCACCG--------TAGCA-3′	1%
	-8	5′-GATCAGGGACCAGTCCACCGTA--------GCA-3′	1%
E5	WT	5′-AAAAATCTTCGCCAGATCCTAGGGGAGGAGCAG-3′	59%
	-1	5′-AAAAATCTTCGCCAGATCCTAG-GGAGGAGCAG-3′	22%
	-1	5′-AAAAATCTTCGCCAGATCCTA-GGGAGGAGCAG-3′	13%
	+1	5′-AAAAATCTTCGCCAGATCCTAGNGGGAGGAGCAG-3′	1%
	-2	5′-AAAAATCTTCGCCAGATCCTA--GGAGGAGCAG-3′	1%

c

sgRNA	Single colonies	Precise edit	Hetero/ homo
E2-1	2/12	16.7	1/1
E2-2	2/5	40	0/2
E5	6/7	85.7	2/4

sFigure 1. Base-editing efficiency and mutation profiles at the IGF1R locus.

Related to Figure 2

(**a**) Proportion of nucleotide substitutions originating from cytosine residues at each position within the three target sequences.

(**b**) Summary of indel frequencies detected at each target site following BE4 or Cas9 editing.

(**c**) Summary of heterozygous (hetero) and homozygous (homo) modifications identified in single-cell colonies generated by BE4 editing.

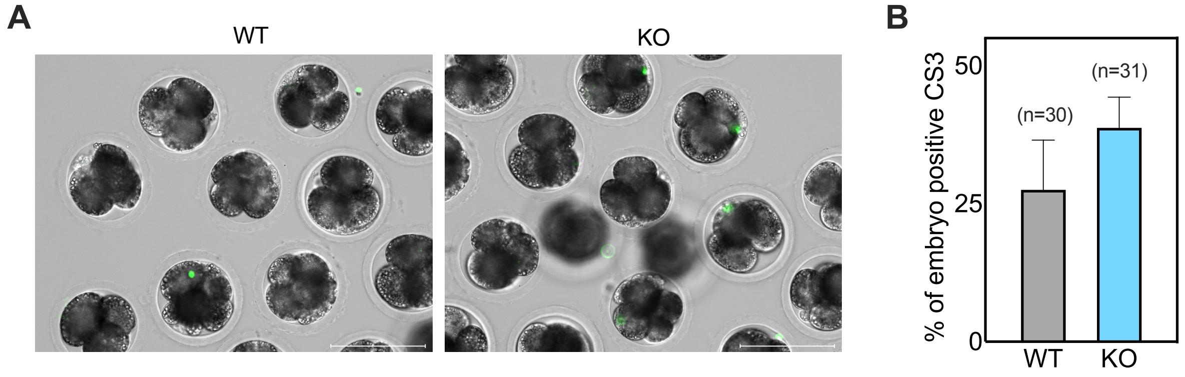

sFigure 2. Increased apoptotic activity in IGF1R-deficient blastocysts. Related to Figure 4.

(**A**) Representative images of live caspase-3/7 (CS3) staining (green) in wildtype control (WT) and treated 4-cell stage embryos at D3. Scale bar: 275 μm.

(**B**) Percentage of CS+ embryos in wild-type (WT) and IGF1R-knockout (KO) blastocysts. Embryos were stained using a live caspase-3/7 detection reagent for 30 mins. Average CS+ cells numbers between WT (n=30) and KO (n=31) blastocysts were compared using an unpaired t-test.

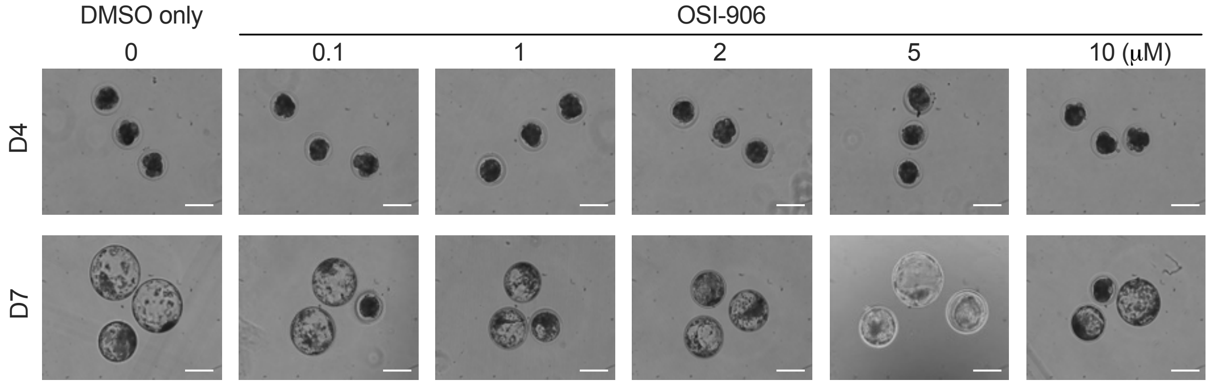

sFigure 3. Experimental design for OSI-906 dose titration in parthenogenetic embryos. Related to Figure 5

Representative image of the embryos following OSI-906 dose titration. Scale bar, 100 μm.

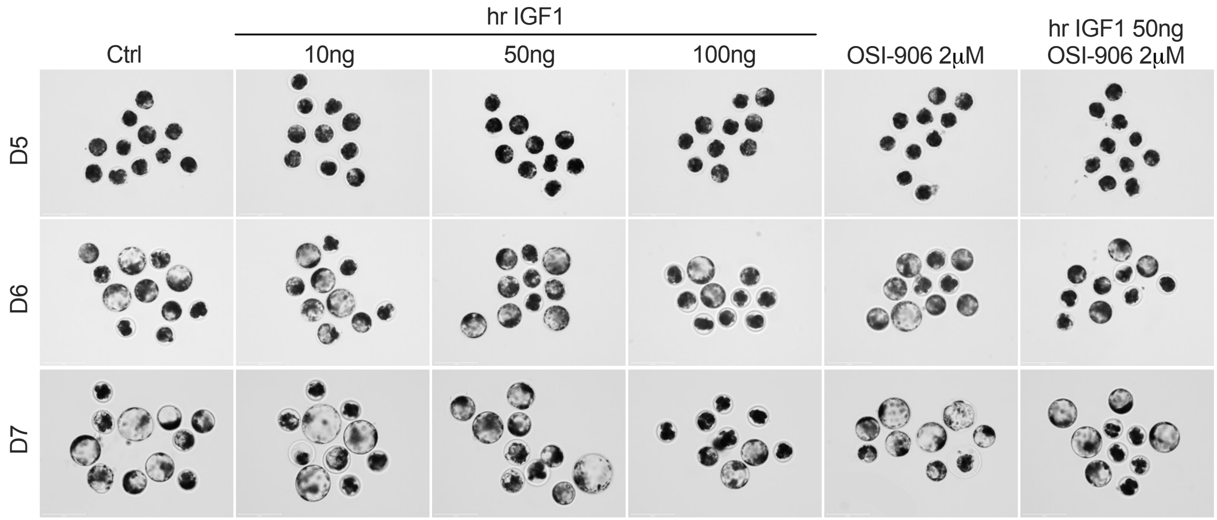

sFigure 4. Quantification of blastocyst formation kinetics following pharmacologic perturbation. Related to Figure 6

Developmental progression of embryos shown in Figure 6A, quantified as the percentage of embryos reaching the blastocyst stage over time. Scale bar, 275 μm.

Supplementary Tables

sTable 1. The sgRNA sequence of IGF1R

Name	sgRNA sequence 5’-3’	
E2-1	CTACCAGCAGCTGAAGCGCC	TGG
E2-2	GGGACCAGTCCACCGTAGAG	AGG
E5	TCTTCGCCAGATCCTAGGGG	AGG

sTable 2. The genotyping primer sequence of *IGF1R*

Gene	Forward/ Reverse primer sequence 5’-3’
IGF1R	GAGGGGTGGGCGAAGACTGAGT/AAAAACAGGAGCCCCCACAGCG/<br>TGGAGGAGAAGCCGCTGTGTGA/TTTCTGGCAGCGGTTCGTGGTC

sTable 3. RT-qPCR primers

Gene	Forward primer (5'-3')	Reverse primer (5'-3')
RN18S	ACAAATCGCTCCACCAACTAAGA	CGGACACGGACAGGATTGAC
ACTB	GTGGACATCAGGAAGGACCTCTA	ATGATCTTGATCTTCATGGTGCT
OCT3/4	GCTGGAGCCGAACCCCGAGG	CACCTTCCCAAAGAGAACCCCCAAA
SOX2	AACAGCCCAGACCGAGTTAA	GTTGTGCATCTTGGGGTTCT
GATA6	ATCACCATCACCACCCAAGT	CGCGACTCTGTAGACTGTGC
SOX17	TGGTTGAATCTTGAGGTCTGC	CAGGGTGTAGGTGTGTGATGA
CDX2	TCGCCCACAAATGTTCACCAAC	TCCAACCGCACCTGTCTTTACC
GATA3	AAAGAGAGAGAGACGGAGAGAG	CGAGGAGCAGAGAGGAGAA
BAX	TTGCTTCAGGGTTTCATCCA	GAGACACTCGCTCAACTTCTT
BCL2	GGAGGATTGTGGCCTTCTTT	GTTCAGGTACTCAGTCATCCAC
CASPASE3	AGAACTCTAACTGGCAAACCC	ACGCCATGTCATCTTCAGTC
CASPASE8	TTTGTCTGCTGCATCCTCTC	GCACTTCGAACCAGTGAAATAAG
CCND1	GCCTCGAAGATGAAGGAGACCATC	TCCATTTGCAGCAGCTCGTC
CDKIB	CCAACTCAGAGGACACACATT	GGCAGGTCGCTTCCTTATC

sTable 4. Antibody information

Antibody	Manufacturer	Species	Dilution ratio	Application
SOX2	Invitrogen, 14981180	Rat	1:100	IF
GATA6	Santa cruz, sc-518050	Mouse	1:100	IF
CDX2	Abcam, ab76541	Rabbit	1:200	IF
IGF1R	Invitrogen, PA5-79444	Rabbit	1:100	IF
Caspase-3/7	Invitrogen, C10423	-	1:400	Live staining

sTable 5. Summary of the CRISPR-induced mutations

Groups	Target site	Nature of mutation	Mutation rate	KO efficiency
CRISPR/Cas9	Exon 2-1	Indels	+1 (10%), -2 (3%)	13 %
	Exon 2-2	Indels	+1 (35%), -8 (2%)	37 %
	Exon 5	Indels	-1 (35%), +1 (1%), -2 (1%)	37 %
BE4	Exon 2-1	C to T	17.3 %	17.9 %
	Exon 2-2	C to T	56.4 %	48.5 %
	Exon 5	C to T	73.9 %	45.4 %

+ insertion, - deletion
